# Identifying low density lipoprotein cholesterol associated variants in the *Annexin A2* (*ANXA2*) gene

**DOI:** 10.1016/j.atherosclerosis.2017.04.010

**Published:** 2017-06

**Authors:** Roaa Hani Fairoozy, Jackie Cooper, Jon White, Claudia Giambartolomei, Lasse Folkersen, S. Goya Wannamethee, Barbara J. Jefferis, Peter Whincup, Yoav Ben-Shlomo, Meena Kumari, Mika Kivimaki, Andrew Wong, Rebecca Hardy, Diana Kuh, Tom R. Gaunt, J.P. Casas, Stela McLachlan, Jackie F. Price, Aroon Hingorani, Anders Franco-Cereceda, Thomas Grewal, Anastasia Z. Kalea, Steve E. Humphries, UCLEB consortium

**Affiliations:** a Cardiovascular Genetics, BHF Laboratories, Institute of Cardiovascular Science, University College London, London, United Kingdom; b UCL Genetics Institute, Department of Genetics, Environment and Evolution, University College London, London, United Kingdom; c Division of Psychiatric Genomics, Department of Psychiatry and Friedman Brain Institute, Icahn School of Medicine at Mount Sinai, One Gustave L. Levy Place, New York, NY 10029, USA; d The Karolinska Institute, Stockholm, Sweden; e Department of Bioinformatics, Technical University of Denmark, Lyngby, Denmark; f UCL Department of Primary Care & Population Health, UCL Institute of Epidemiology, University College London, London, United Kingdom; g Population Health Research Institute, St George's University of London, Cranmer Terrace, London, United Kingdom; h School of Social and Community Medicine, University of Bristol, Bristol, United Kingdom; i Institute for Social and Economic Research, University of Essex, Colchester, United Kingdom; j Department of Epidemiology & Public Health, UCL Institute of Epidemiology & Health Care, University College London, London, United Kingdom; k MRC Unit for Lifelong Health and Ageing, London, United Kingdom; l MRC Epidemiology Unit, Institute of Metabolic Science, Addenbrooke's Hospital, Cambridge, United Kingdom; m Farr Institute of Health Informatics, University College London, London, United Kingdom; n Centre for Population Health Sciences, The University of Edinburgh, Edinburgh, United Kingdom; o Genetic Epidemiology Group, Institute of Cardiovascular Science, University College London, London, United Kingdom; p Cardiothoracic Surgery Unit, Department of Molecular Medicine and Surgery, Karolinska Institutet, Stockholm, Sweden; q Faculty of Pharmacy, University of Sydney, Sydney, NSW 2006, Australia

**Keywords:** Annexin A2, Proprotein convertase subtilisin/kexin type-9, Low-density lipoprotein cholesterol-receptor, Low-density lipoprotein cholesterol, Single nucleotide polymorphism, Coronary heart disease, AnxA2, annexin A2, PCSK9, proprotein convertase subtilisin/kexin type-9, LDLR, low-density lipoprotein cholesterol-receptor, LDL-C, low-density lipoprotein cholesterol, NPHSII, Second-Northwick-Park Heart Study, UCLEB, UCL-LSHTM-Edinburgh-Bristol consortium, CHRD, cysteine-histidine-rich domain of PCSK9, FAIRE, formaldehyde assisted isolation of regulatory elements, CTCF, CTC-binding factor

## Abstract

**Background and aims:**

Annexin-A2 (AnxA2) is an endogenous inhibitor of proprotein convertase subtilisin/kexin type-9 (PCSK9). The repeat-one (R1) domain of AnxA2 binds to PCSK9, blocking its ability to promote degradation of low-density lipoprotein cholesterol-receptors (LDL-R) and thereby regulate low-density lipoprotein cholesterol (LDL-C) levels. Here we identify variants in *ANXA2* influencing LDL-C levels and we determine the molecular mechanisms of their effects.

**Results:**

The *ANXA2* single nucleotide polymorphism (SNP) genotype-phenotype association was examined using the Second-Northwick-Park Heart Study (NPHSII) (n∼2700) and the UCL-LSHTM-Edinburgh-Bristol (UCLEB) consortium (n∼14,600). The *ANXA2*-R1 domain coding-SNP rs17845226 (V98L) associated with LDL-C, homozygotes for the minor allele having ≈18.8% higher levels of LDL-C (*p* = 0.004), and higher risk of coronary heart disease (CHD) (*p* = 0.04). The SNP is in modest linkage disequilibrium (r^2^ > 0.5) with two intergenic SNPs, rs17191344 and rs11633032. Both SNPs showed allele-specific protein binding, and the minor alleles caused significant reduction in reporter gene expression (≈18%, *p* < 0.001). In the expression quantitative trait loci (eQTL) study, minor allele homozygotes have significantly lower levels of *ANXA2*-mRNA expression (*p* = 1.36 × 10^−05^).

**Conclusions:**

Both rs11633032 and rs17191344 SNPs are functional variants, where the minor alleles create repressor-binding protein sites for transcription factors that contribute to reduced *ANXA2* gene expression. Lower AnxA2 levels could increase plasma levels of PCSK9 and thus increase LDL-C levels and risk of CHD. This supports, for the first time in humans, previous observations in mouse models that changes in the levels of AnxA2 directly influence plasma LDL-C levels, and thus implicate this protein as a potential therapeutic target for LDL-C lowering.

## Highlights

• Human genome data, bioinformatics studies used to nominate *ANXA2* candidate variants.
• Molecular genetics analysis used to identify functional variants in *ANXA2* gene.
• Finding common functional variation in *ANXA2* has implications for health.

## 1 Introduction

Hypercholesterolemia is a major risk factor for atherosclerosis and coronary heart disease (CHD), most often caused by an individual having a greater than average number of common lipid-raising SNPs. The Global Lipids Genetics consortium (2013) has identified 157 novel loci associated with lipid levels, 15 of which are known to influence plasma levels of Low Density Lipoprotein cholesterol (LDL-C) [1]. PCSK9 binds to the epidermal growth factor domain A (EGF-A) of the LDL-R via its catalytic domain either intracellularly or at the cell surface [2]. Once the PCSK9≡LDL-R complex is formed, it is internalised by endocytosis and degraded [3], [4]. Gain-of-function mutations in *PCSK9* strongly promote LDL-R degradation and lead to FH, whereas loss-of-function mutations of *PCSK9* are unable to enhance LDL-R downregulation and therefore result in lower levels of LDL-C [5]. This suggests that lowering PCSK9 will protect against atherosclerosis and CAD.

AnxA2 has been identified in animal and cellular models as an endogenous inhibitor of PCSK9 and thus influences LDL-Receptor and plasma cholesterol levels [6], [7], [8]. AnxA2 is widely expressed, and in mice, high AnxA2 levels are found in the lung, pancreas, colon, ileum and adrenal tissues. In contrast, spleen, testis, kidney and liver express low AnxA2 levels [8]. AnxA2 belongs to the conserved annexin family of phospholipid and calcium-binding proteins. AnxA2 exists as a monomer, yet the majority of AnxA2 forms a heterotetramer with the S100 protein p11 (S100A10) both in intra- and extracellular locations [9], [10]. Inside cells, AnxA2 regulates a spectrum of functions related to membrane organization and trafficking [9], [11], [12]. In plasma, in particular on the surface of endothelial cells, the AnxA2/p11 complex is involved in vascular fibrinolysis [9], [10]. In addition, AnxA2 has several other extracellular AnxA2 activities [13]. Most relevant to this study, AnxA2, either as monomer or complexed with p11, is involved in cholesterol metabolism through the binding of its R1-domain to the cysteine-histidine-rich domain (CHRD) of PCSK9 at the cell surface, which inhibits PCSK9-mediated degradation of LDL-R. This helps to maintain LDL-R levels at the cell surface with the subsequent greater clearance of LDL-C [6], [8]. An *in vitro* study reported that a mutation Q554E in the CHRD of PCSK9 increased the binding affinity between PCSK9 and AnxA2, which in turn lead to a loss-of-function of PCSK9 towards LDL-R degradation [6]. This suggested an involvement of AnxA2 in the regulation of LDL-C levels, and subsequent *in vivo* studies in AnxA2 knockout mice identified higher levels of plasma PCSK9 and LDL-C, which correlated with a reduction in LDL-R protein levels, mostly in extrahepatic tissues [8]. Moreover, adenoviral AnxA2 overexpression in mouse liver significantly increased hepatic LDL-R levels [8]. Therefore, we hypothesized that a mutation in the *ANXA2* R1-domain could also affect LDL-C levels.

The *ANXA2* locus is located on chromosome 15q22.2 and consists of 13 exons [14], and its expression is regulated at both the transcriptional and translational levels [9]. The R1-domain of *ANXA2* is encoded by exons 4–6, which has eight reported SNPs including one missense variant rs17845226, which changes Valine to Leucine at position 98. This SNP was selected for further study because it has been validated by HapMap and the 1000 Genome Project, and is the only SNP that has a minor allele frequency (MAF) ≥ 0.05. Also, in a preliminary study including only 43 subjects, this SNP was implicated to affect circulating PCSK9 levels [8], but a thorough analysis of its association with LDL-C and CHD in larger cohorts has not yet been performed. To elucidate the molecular mechanism behind the effect seen, the linkage disequilibrium (LD) of this SNP with others at the locus was examined, and bioinformatics and *in vitro* functional assays were used to determine the likely functional SNPs at this locus.

## 2 Materials and methods

### 2.1 Study cohorts

The Second-Northwick-Park Heart Study (NPHSII) consists of 3052 (with DNA available n∼2700) healthy middle-aged men (50–61 years) who were recruited in 1989 from nine general medical practices in the United Kingdom (UK) and followed for up to 15 years. The UCL-LSHTM-Edinburgh-Bristol (UCLEB) consortium consists of 30,000 participants from 12 well-established UK studies (participants are almost exclusively of European ancestry). Further details of studies background can be found in Supplementary Materials.

### 2.2 Genotyping and statistics

The *ANXA2* SNPs rs17845226 and rs17191344 were genotyped in the NPHSII study using Applied Biosystems TaqMan SNP Genotyping Assay. The assay mix was added over 5 ng dry DNA and thermocycled as per the manufacturer's instructions, and fluorescence detected with ABI 7900HT. Statistical analyses for both NPHSII and UCLEB are explained in details in Supplementary Materials.

### 2.3 Bioinformatics

Multiple algorithms were used to predict the impact of missense mutations (*ANXA2*-R1 rs17845226 SNP V98L) on protein structure and function: Sorting Intolerant Form Tolerant (SIFT), Polyphen-2 V2, and Mutation Assessor V3 [15]. The 1000 Genomes Project data and the Broad Institute's HaploReg V4.1 [16], [17], [18] were used to identify variants in strong (r^2^ ≥ 0.8) and modest (r^2^ ≥ 0.4) linkage disequilibrium (LD) with the *ANXA2*-coding SNP rs17845226. These variants were examined for regulatory annotations from the ENCODE Project [19], [20] and the RoadMap Epigenomics data [21], To visualize variant location, the UCSC Genome Browser was used [22]. The ElDorado tool (Genomatix Software GmbH, Germany) [23] was used to select variants with only strong motif changes (thresholds had core similarity of 1 and matrix similarity of >0.8). Further details of bioinformatics analyses can be found in Supplementary Materials.

### 2.4 Electrophoretic mobility shift assay (EMSA)

EMSAs were used to investigate the effect of variants' genotype on DNA-protein binding. Nuclear extract for EMSA was obtained from hepatocarcinoma Huh7 cells as described in Ref. [24]. Biotinylated allele-specific probes for the three selected SNPs were incubated with a Huh7 cell nuclear extract (probe sequences in Supplementary Table 1). EMSA was performed as described in Ref. [25].

### 2.5 Luciferase reporter assay

To generate luciferase-constructs, The *ANXA2* intergenic SNP sequences encompassing the SNP alleles [rs17191344 A > G (776 bp) and rs11633032 G > A (593 bp) (primer sequences are shown in Supplementary Table 4) were individually inserted into the enhancer site of the pGL3-promoter luciferase reporter vector (Promega) after the SV40 polyadenylation signal according to the manufacturer's instructions. Both reference allele and alternative allele luciferase-constructs were transfected into Huh7 cells along with the Renilla luciferase pRL-TK as co-transfectant control. The firefly and renilla luciferase activity was detected using Promega's Dual-Luciferase Reporter Assay System according to the manufacturer's instructions.

## 3 Results

### 3.1 Bioinformatics analysis

**Fig. S1:**
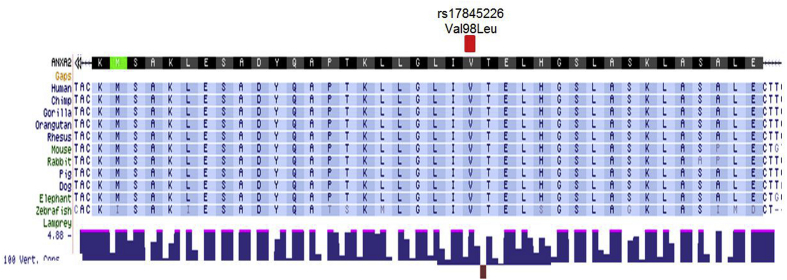


The R1-domain of *ANXA2* is encoded by exons 4–6, which has eight reported SNPs, including one missense variant rs17845226. The *ANXA2*-R1 rs17845226 SNP is located in exon 6 of the gene and causes a Valine/Leucine amino acid change; the MAF is 12% in European populations (1000 Genomes Project Phase 3). Although the altered amino acid (Valine) is highly conserved in vertebrate species (Supplementary Fig. 1), the change is predicted to be non-pathogenic by the SIFT, Polyphen-2, and MutationTaster predicting tools.

**Fig. 1:**
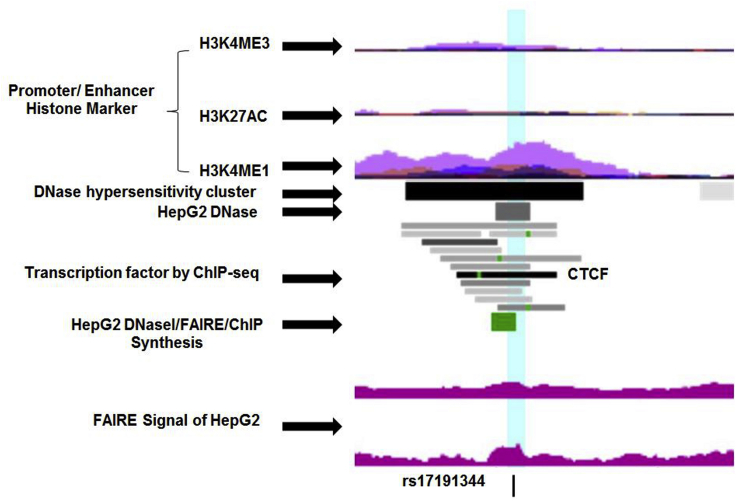
Genome-wide maps of chromatin state of *ANXA2* rs17191344 SNP. Schematic representation of the *ANXA2* rs17191344 chromatin status (shttps://genome-euro.ucsc.edu). The area of interest is highlighted in light-blue. Promoter/enhancer histone marker includes seven cell lines (GM12878, H1-hESC, HSMM, HUVEC, K562, NHEK, and NHLF). FAIRE, formaldehyde assisted isolation of regulatory elements. (For interpretation of the references to colour in this figure legend, the reader is referred to the web version of this article.)

**Fig. S2:**
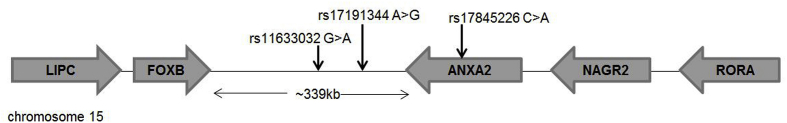


**Fig. S3:**
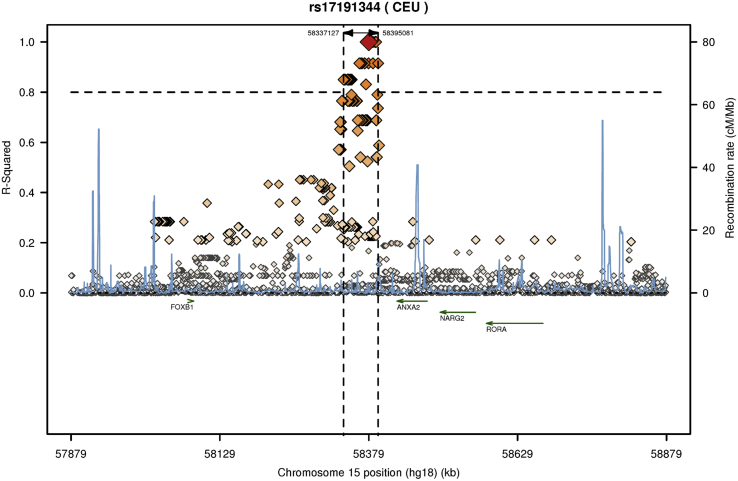


The *ANXA2*-R1 rs17845226 SNP has modest LD (r^2^ ≥ 0.4) with 34 SNPs, all located downstream of the *ANXA2* gene-coding region in the long intergenic region between two genes, *FOXB* and *ANXA2* on chromosome 15, and near the *RORA* and *LIPC* loci, which play roles in lipid metabolism and atherosclerosis (Supplementary Fig. 2). Out of 34 SNPs, the rs17191344 SNP (r^2^ = 0.45, MAF = 16%) has the strongest regulatory profile, where the SNP is highly conserved and has strong enhancer signs in 13 tissues including the liver. The SNP is located in an open chromatin region, where the markers of DNAase I, FAIRE, and transcription factor binding are strong (Fig. 1). Both ENCODE and ElDorado data show that the G allele of the rs17191344 SNP creates a binding site for CTCF, and this SNP also changes a YY1 binding motif. The YY1 transcription factor can associate with CTCF and regulate gene expression [26]. The rs17191344 SNP has strong LD (r^2^≥0.8) with 66 SNPs, all in the intergenic region (Supplementary Fig. 3), of which SNPs rs11633032 and rs12900101 (MAF = 17%) are predicted to bind to regulatory transcription factors as shown in the ElDorado data. These two SNPs are also in modest LD with the ANXA2-coding SNP rs17845226 (r^2^ = 0.40 and 0.41, respectively).

### 3.2 Genotype-phenotype association

#### 3.2.1 Association of ANXA2 SNPs rs17845226 and rs17191344 with lipid traits and CHD in NPHSII

The association of rs17845266 and rs17191344 with LDL-C and CHD was investigated in the NPHSII cohort. Baseline study characteristics are summarized in Supplementary Table 5. The minor allele frequency of rs17845266 in NPHSII was 0.13 while the rs17191344 minor allele frequency was 0.145, both similar to that seen in European populations.

rs17845226 showed a significant association with LDL-C and CHD under both modes of inheritance (recessive and additive). Table 1 shows that individuals who are homozygous for the minor allele (A) had significantly higher levels of total cholesterol (TC) ≈8.4% and LDL-C ≈18.8% (*p* = 0.01 and 0.004, respectively), and had a significantly higher risk of CHD (HR (95% CI): 2.17 (1.03–4.60), *p* = 0.04)). The most likely cause of the observed CHD association is that it is due to these subjects having a higher level of LDL-C, and when the CHD association was adjusted for LDL-C, the effect was no longer statistically significant. This suggests that the main mechanism for this SNP to be influencing risk of CHD is via its effect on LDL-C levels.

**Table 1:** An association between rs17845226 and rs17191144 genotype and lipid risk factors and CHD risk in the NPHSII cohort.

Genotype	rs17845226 (Val98Leu)					rs17191344				
	CC (n = 1868)	CA (n = 561)	AA (n = 34)	*p*-value^a^	*p*-value^b^	AA (n = 1956)	AG (n = 659)	GG (n = 57)	*p*-value^a^	*p*-value^b^
TC (mmol/L)	5.73 (1.00)	5.80 (1.04)	6.21 (1.05)	0.014	0.007	5.72 (1.01)	5.74 (1.03)	5.96 (1.04)	0.205	0.085
LDL-C (mmol/L)	3.08 (0.99)	3.16 (1.04)	3.66 (0.94)	0.006^d^	0.003	3.09 (1.00)	3.09 (1.03)	3.38 (1.03)	0.302	0.051
HDL-C (mmol/L)	0.81 (0.24)	0.80 (0.26)	0.74 (0.23)	0.456	0.201	0.81 (0.24)	0.79 (0.25)	0.81 (0.26)	0.494	0.786
TG (mmol/L)^c^	1.74 (1.25–2.54)	1.7 (1.22–2.65)	1.74 (1.22–2.91)	0.970	0.648	1.73 (1.24–2.53)	1.76 (1.25–2.74)	1.62 (1.12–2.56)	0.465	0.604
**CHD risk by genotype**										
No. without CHD (%)	1672 (75.8)	507 (23.0)	26 (1.2)	–	–	1749 (73.5)	585 (24.6)	45 (1.9)	–	–
No. with CHD (%)	196 (76.0)	54 (20.9)	8 (3.1)	–	–	207 (70.7)	74 (25.3)	12 (4.1)	–	–
HR (95% CI) Model 1	1.00	0.93 (0.68–1.26)	2.17 (1.03–4.60)	0.578^e^	0.043	1.00	1.10 (0.83–1.44)	1.86 (1.02–3.41)	0.113	0.052
HR (95% CI) Model 2	1.00	0.84 (0.59–1.20)	1.25 (0.49–3.20)	0.573	0.60	1.00	0.98 (0.72–1.34)	1.29 (0.60–2.79)	0.787	0.504

^d and e^ Deviation from a dominant model *p* = 0.051.

^d and e^ Deviation from a dominant model *p* = 0.021.

Model 1: age and practice adjusted; Model 2: adjusted for age, practice and LDL-C.

a *p*-value according to additive model.

b *p*-value according to recessive model.

c Median [IQR].

**Fig. S4:**
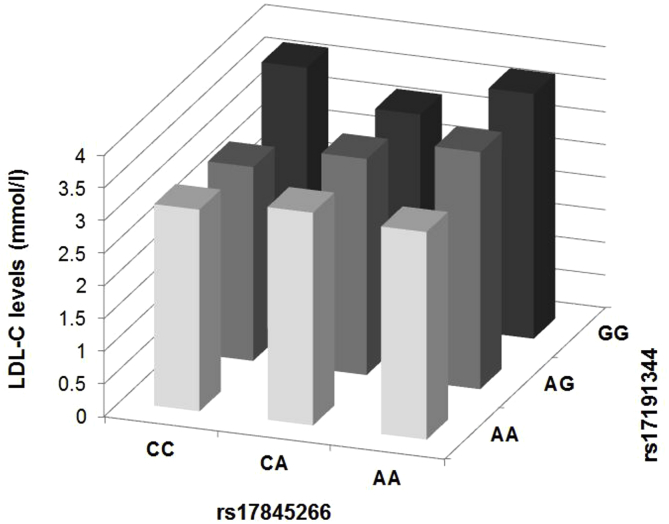


The modest LD SNP rs17191344 shows the same trend as the lead coding SNP (Table 1), where individuals who carried two copies of the minor allele have higher levels of LDL-C (*p* = 0.05) and a higher risk of CHD [HR (95% CI) = 1.86 (1.02–3.41), (*p* = 0.05). Table 2 and Supplementary Fig. 4 show the combined genotype association of rs17191344 and rs17845226, where LDL-C levels increase per minor allele of both SNPs. The individuals who have two copies of the minor allele of both SNPs (G and A respectively) had significantly higher levels of LDL-C (*p* = 0.007) and over two-fold higher risk of CHD [HR (95% CI) = 2.75 (1.18–6.39), (*p* = 0.019)]. Stepwise models indicated that only those with two copies of the minor allele of both SNPs had significantly raised levels for the lipids: TC [β(se) = 0.482 (0.22), r^2^ = 0.002, *p* = 0.029] and LDL-C [β(se) = 0.649, r^2^ = 0.004, *p* = 0.008] (Supplementary Table 6). This confirms the result from Table 2, where only the GG/AA group has significantly different levels from the other groups for these lipid traits. For CHD, the GG/CC group had significantly higher risk compared to the other groups [HR (95% CI) = 6.53 (1.62–26.27), *p* = 0.008].

**Table 2:** The rs17191344 and rs17845226 SNPs combined genotype association with lipids traits and CHD in the NPHSII cohort.

rs17191344	rs17845226	Lipids					CHD			
		N	TC (mmol/L)	*p*-value	LDL-C (mmol/L)	*p*-value	N	% (N) with CHD	HR (95% CI)	*p*-value
AA	CC	1658	5.73 (1.00)	–	3.09 (0.99)	–	1658	10.7 (177)	1.00	–
	CA	75	5.81 (1.05)	0.49	3.26 (1.06)	0.21	75	6.7 [5]	0.53 (0.21–1.33)	0.18
	AA	3	5.57 (0.45)	0.78	3.18 (0.72)	0.88	3	0 (0)	–	–
AG	CC	137	5.67 (0.97)	0.49	2.97 (1.00)	0.21	137	12.4 [17]	1.32 (0.79–2.20)	0.29
	CA	443	5.78 (1.04)	0.33	3.13 (1.04)	0.48	443	10.6 (47)	1.03 (0.74–1.43)	0.88
	AA	9	6.32 (0.92)	0.08	3.63 (1.08)	0.15	9	22.2 [2]	3.17 (0.78–12.92)	0.11
GG	CC	4	6.18 (0.36)	0.38	3.72 (0.77)	0.28	4	50.0 [2]	8.60 (2.09–35.34)	0.003
	CA	23	5.90 (0.94)	0.43	3.22 (1.04)	0.56	23	13.0 [3]	1.35 (0.43–4.26)	0.604
	AA	21	6.22 (1.17)	0.025	3.75 (0.96)	0.007	21	28.6 [6]	2.75 (1.18–6.39)	0.019

Adjusted for age and practice.

There were no significant SNP × SNP interactions. TC *p* = 0.75, LDL-C *p* = 0.83, CHD *p* = 0.27.

#### 3.2.2 Association of ANXA2 intergenic SNPs with lipid traits and CHD in the UCLEB consortium

The UCLEB consortium, comprising ∼14,600 subjects from the UK general population, was used for replication. Study characteristics are summarized in Supplementary Table 7. We were unable to impute rs17191344, but two SNPs rs11633032 (G > A) and rs12900101 (C > G) [having a strong LD (r^2^ = 0.98) with rs17191344] had a high average value of imputation genotype data from the Metabochip (r^2^ = 0.63) (Supplementary Table 8).

**Fig. S5:**
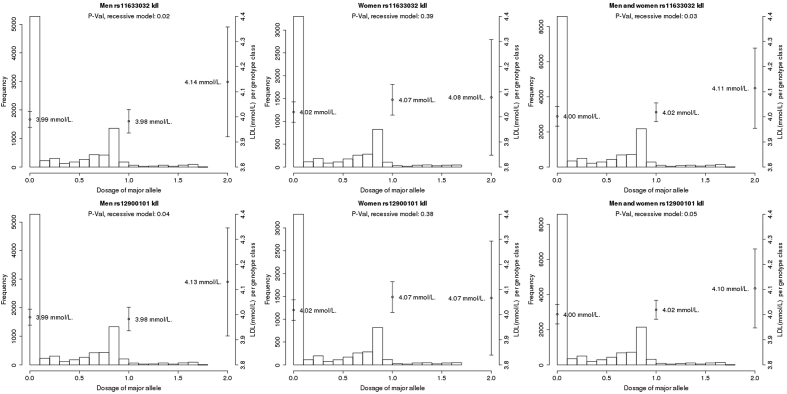


As shown in Supplementary Table 9 and Supplementary Fig. 5, the minor alleles of rs11633032 and rs12900101 were associated with significantly higher levels of LDL-C in men [effect size = 0.21 mmol/L, *p* = 0.018 and 0.19 mmol/L, *p* = 0.036 respectively for the recessive model], but not in women, which may partly be due to different sample sizes between the genders. To assess whether there the effect is different between the sexes, we tested the estimated difference between sexes, and no difference was found (Supplementary Table 10). Overall, the minor alleles of rs11633032 and rs12900101 were associated with significantly higher levels of LDL-C [effect size = 0.16 mmol/L, *p* = 0.029 and 0.143 mmol/L, *p* = 0.048 respectively for the recessive model] with similar effects in both genders (Supplementary Table 9). This result confirms the results seen above in the NPHSII subjects, where subjects are men only.

### 3.3 Allele-specific protein binding of ANXA2 intergenic SNPs in Huh7 cells

**Fig. 2:**
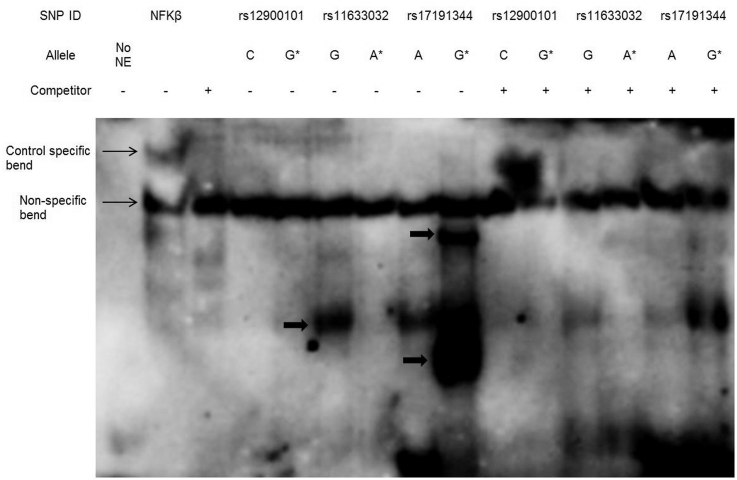
DNA binding properties of *ANXA2*-intergenic SNPs. Conventional EMSA analysis of the *ANXA2*-intergenic SNPs (rs12900101, rs11633032 and rs17191344). The major allele of rs11633032 has allele-specific binding that is competed out by the allele competitor probe. The minor allele of rs17191344 has allele-specific binding at two positions, which are competed out by the allele competitor probe. Allele specific bands are indicated by arrows and (*) indicates minor allele.

EMSA was performed to determine whether the three *ANXA2*-intergenic SNPs within potential regulatory elements were able to affect DNA-protein interactions. Two SNPs, rs11633032 and rs17191344, demonstrated differential protein binding by allele (Fig. 2). The rs11633032 major G allele bound to proteins or complexes of proteins, whereas the risk A allele did not show allele-specific protein binding. In contrast, the risk G allele of the rs17191344 SNP bound strongly to proteins.

**Fig. S6:**
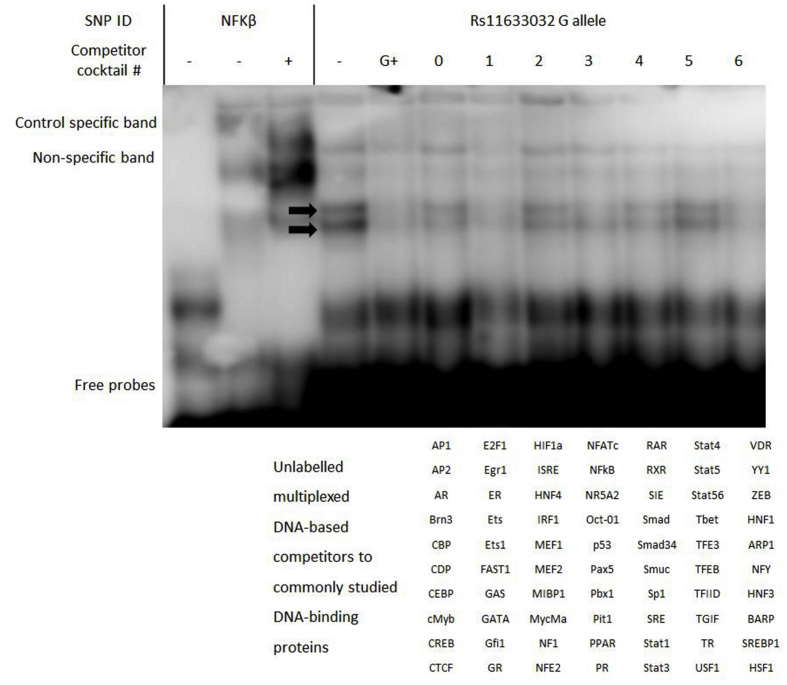


MC-EMSA was performed to characterise the DNA-protein interaction for the major G allele of the rs11633032 SNP. The results showed that the G allele specific-bands were competed out by cocktail 1 (Supplementary Fig. 6A). Then, when each competitor of cocktail 1 was run individually, the G allele specific-bands were competed out by addition of the GATA and Egr1 consensus sequences (Supplementary Fig. 6B).

**Fig. S7:**
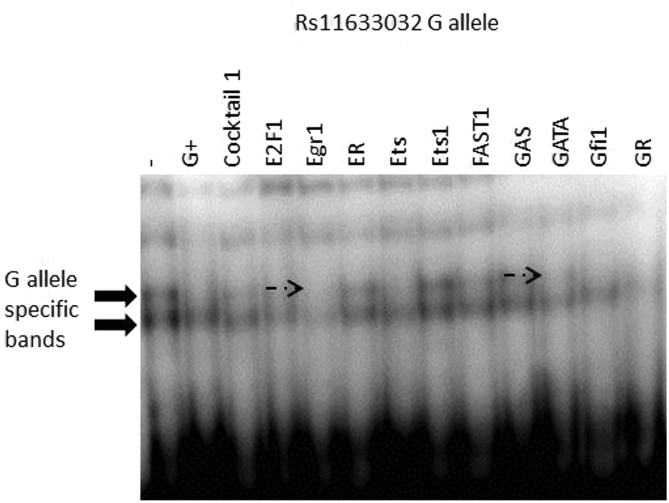


The bioinformatics analysis for the rs17191344 SNP suggests that the risk allele of the SNP is a site for CTCF protein binding. Comparing the CTCF-binding motif to the genomic sequence around the rs17191344 revealed that they matched up well, and the presence of the risk G allele of the SNP strengthened the binding motif (Supplementary Fig. 7A). In EMSA, the G allele of the SNP was competed out with 11 different isoforms of CTCF [27]. The results showed that the rs17191344 G allele specific bands were competed out by at least three isoforms of CTCF (Supplementary Fig. 7B), suggesting that CTCF is the protein that binds to the sequence around the G allele of the SNP.

### 3.4 Effect of rs17191344 and rs11633032 on reporter gene expression

**Fig. 3:**
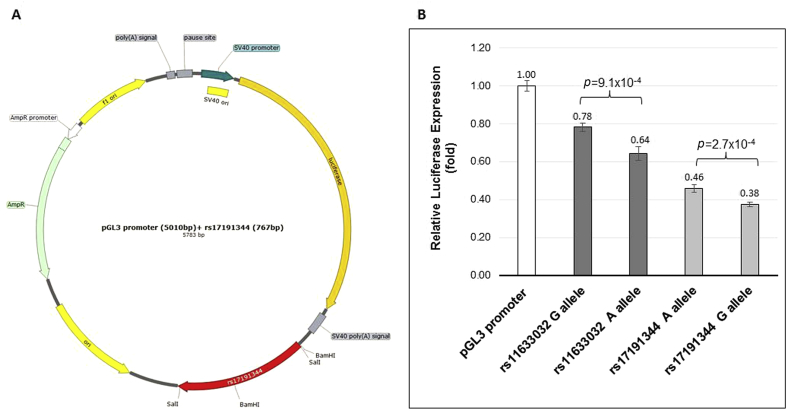
*ANXA2*-intergenic SNPs luciferase activity in the Huh7 cell line. (A) A map of the pGL3-promoter constructs showing the location of the added SNP fragment of rs11633032 (585 bp) and rs17191344 (767 bp). (B) The luciferase reporter assays showing relative expression of *ANXA2*-intergenic SNPs-luciferase-enhancer-constructs (sequence has SNP of interest inserted into enhancer site of the vector) relative to the control (pGL3-promoter vector).

Luciferase reporter assays were performed to assess whether the rs17191344 and rs11633032 SNPs genotype affect gene expression. The *ANXA2* SNPs rs11633032 (593 bp) and rs17191344 (776 bp) fragments containing either allele of the SNP was inserted downstream of the luciferase gene in the pGL3-promoter vector (Fig. 3A). The inserted fragment in the pGL3-promoter vector resulted in a decrease in expression compared to the control vector for both alleles of rs11633032 and rs17191344 (Fig. 3B). However, the presence of minor alleles caused approximately 18% further significant decreases of gene expression in rs11633032 (*p* = 9.1 × 10^−4^) and rs17191344 (*p* = 2.7 × 10^−4^). This suggests that the sequences around rs11633032 and rs17191344 are sites for repressor protein binding.

### 3.5 Expression quantitative trait loci (eQTL) analysis

**Fig. S8:**
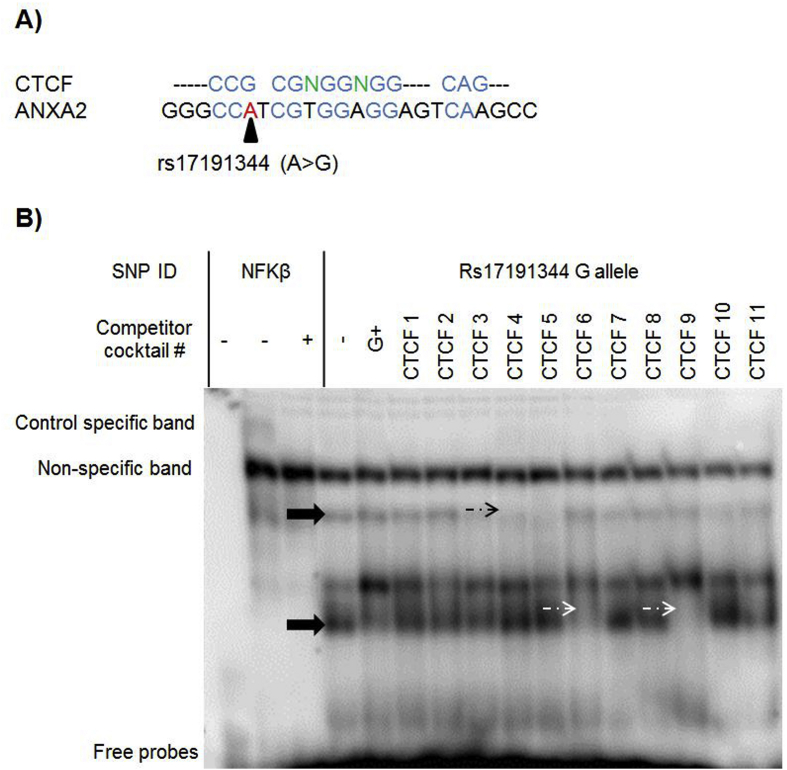


To determine whether *ANXA2* intergenic SNPs were associated with altered gene expression *in vivo*, we first used the publicly available gene expression data set GTEx. The four genes (*FOXB, ANXA2, RORA* and *LIPC*) near the SNPs were tested in GTEx, but no significant association was found between the SNPs genotype and gene expression in the liver, whole blood or coronary artery (Supplementary Fig. 8). However, subjects with one copy of the risk allele for the rs11633032 had lower expression of *ANXA2* in all three tissues (effect size −0.062 and −0.039, −0.20 respectively), but this effect was not statistically significant (*p* > 0.05) due to the small sample size, with only three-six subjects being homozygous for the minor allele. However this effect is confirmed in the latest GTEx data, which showed the rs11633032 minor allele was associated with significantly reduced *ANXA2* expression in the tibial artery sample (effect size −0.177, *p* = 2.9 × 10^−06^, N = 285) under additive model.

**Fig. S9:**
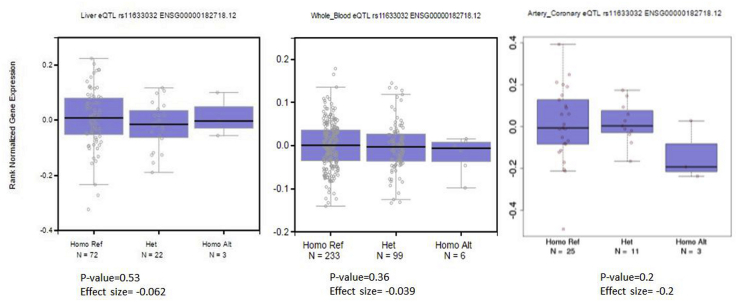


To examine this further, the ASAP database was used. This showed low levels of *ANXA2* expression in liver tissue, with again the rs11633032 SNP risk A allele being associated with reduced expression level of *ANXA2* (*p* = 0.075) (Supplementary Fig. 9), but with no other nearby gene including LIPC. To further verify these findings, we used the publicly available eQTL meta-analysis for lipid-regulation [28] and found the proxy SNP rs9920796 (r^2^ = 0.736) was significantly associated with lower *ANXA2*-mRNA expression levels in blood (Z-score = −4.35, *p* = 1.36 × 10^−05^). Overall, these findings suggest that the *ANXA2*-intergenic SNPs rs11633032 and rs17191344 are sites for repressor protein binding that reduces the expression level of the gene.

## 4 Discussion

Biological studies in mice have recently supported cell culture studies that implicate AnxA2 in the prevention of PCSK9-mediated degradation of LDL-R [6], [8]. AnxA2 mediates this inhibitory effect via the interaction of its R1-domain with the CHRD of PCSK9. Therefore, we hypothesized that a mutation in the *ANXA2* R1-domain could affect LDL-C levels in humans. We looked at SNPs in the R1-domain and the rs17845226 missense variant Val98Leu was selected for further analysis, as this variant has previously been associated with lower circulating PCSK9 levels [8] and because the MAF in the European population is high at 13%. Using genotype-phenotype analysis, we found that this missense mutation had a recessive effect on LDL-C levels. However, *in silico* tools predicted this SNP to be non-pathogenic, suggesting it may simply be acting as a marker for a functional SNP elsewhere at the locus. Using bioinformatics, genotype-phenotype analysis, and evidence from differential protein binding and allele-specific gene expression, we identified two candidate SNPs in the *ANXA2* cis-regulatory region (rs17191344 and rs11633032) also associated with LDL-C and the risk of CHD, and showed that these SNPs affect *ANX2A* gene expression via alterations in transcription factors that bind to alleles of the SNPs. This work identifies for the first time with statistical significance in humans the observations in cell culture and AnxA2 knockout mice that changes in the levels of AnxA2 directly influence plasma LDL-C levels, and thus implicates this protein, and the pathway in which it operates, as a potential therapeutic target for LDL-C lowering.

The effect on LDL-C levels and CHD risk associated with these *ANXA2* variants is recessive, with significantly higher levels seen only in those carrying two copies of the minor allele. If the Leucine variant is indeed less active, the mechanism of this effect may depend on the fact that AnxA2 exists as a dimer with two R1-domains, and thus in an individual heterozygous for the variant, if one of the two alleles carried is less effective, the other is functional and is still able to bind to PCSK9. However, if both inherited alleles are less effective in their ability to interact with PCSK9, this may allow PCSK9 to act on LDL-R. However, the Val98Leu SNP may simply be acting a as marker for SNPs affecting gene expression, and to attempt to disentangle this, we assessed the cholesterol levels and risk of CHD for these individuals with different combinations of genotypes of the Val98Leu SNP rs17845226 with the cis-regulatory SNP rs17191344 showing modest LD (r^2^ = 0.45). It was found that subjects carrying two copies of the minor allele for both SNPs had the highest LDL-C levels and risk of CHD, but, while numbers are small, subjects carrying *either* of the minor alleles had modestly elevated cholesterol levels and CHD risk, suggesting that both the amino-acid change and the intronic SNPs are functional. Although we reported that the intergenic SNPs did not display a significant difference in cholesterol levels between sexes, women appeared to be less affected by these variants. Several studies have suggested that sex and age have an impact on PCSK9 concentration and consequently LDL-C [29].

In our study, bioinformatics was particularly helpful to select candidate SNPs for functional studies. The Val98Leu SNP has modest LD with 34 SNPs, all located downstream of the *ANXA2* gene-coding region in the long intergenic region. Such regions often have a role in gene regulation by interacting with chromatin-modifying complex proteins [30]. Regulation data from ENCODE and RoadMap Epigenomics were used to evaluate these 34 variants for their regulatory potential, and a shortlist of three potentially functional variants identified (rs17191344, rs11633032 and rs12900101).

These predictions were confirmed by *in vitro* assays. Using EMSA, rs17191344 and rs11633032, showed allele-specific protein binding, with a protein binding strongly to the protective G allele of rs11633032, while rs17191344 showed a strong allele-specific binding to the G risk allele. The luciferase reporter assay was used to assess the mechanism and action of the SNPs and evaluate how different protein binding may affect gene expression. It was found that the minor allele of both SNPs rs11633032 and rs17191344 reduced the gene expression. Such lower gene expression in carriers of the risk A allele of rs11633032 was confirmed using *human* expression data from GTEx, ASAP and from the large eQTL meta-analysis. Taken together, these data strongly suggest that transcriptional repressor proteins binding to the sequence around the minor alleles of SNPs rs11633032 and rs17191344 and thus lead to reduced *ANXA2*-mRNA expression and protein levels. When expressed at low levels, all available AnxA2 may be “moped up” by other high-affinity, high-abundance, AnxA2 binding proteins in plasma such as plasminogen or tPA [10] or even membrane phospholipids. This would leave no available AnxA2 to interact with PCSK9, and allow PCSK9 to bind to LDL-R, leading to lower levels of LDL-R receptors in the liver and consequently, increased plasma LDL-C levels.

Several potential transcriptional factors involved in the regulation of AnxA2 expression levels were identified. MC-EMSA suggested that the GATA and Egr1 proteins bind to the sequence around the G allele of rs11633032, and CTCF binds to the sequence in the presence of the G allele of rs17191344. The protein family GATA comprises six members (GATA1 to GATA6) all of which have a highly conserved double zinc finger domain that mediates binding to DNA and to co-factors to regulate gene expression in a highly tissue-restricted fashion. GATA recruits chromatin remodelling complex and mediates either repression or activation of target genes [31], [32]. The Egr1 is a nuclear factor that regulates gene expression in a tissue-restricted manner, through its binding to other regulatory transcription factors [33]. GATA and Egr1 could be part of a chromatin remodelling complex, which as a complex mediates gene expression. The CTCF is considered an insulator element and plays a critical role in transcriptional regulation. There are two possible functions of an insulator in gene regulation. First, it binds to DNA-regulatory sequences in the promoter-proximal regions where it competes for enhancer-bound activators and prevents the activation of downstream promoters [27], [34]. In addition, insulators could be involved in gene regulation by facilitating the formation of separate loop domain structures, which prevent an enhancer on one loop from contact a promoter on a different loop [35], [36]. Despite the identification of abovementioned transcription factors GATA, Egr1 and CTCF to bind AnxA2 mRNA *in vitro*, future work will need to validate their contribution to the regulation of AnxA2 expression levels in hepatic cell lines. In particular, overexpression or silencing of the transcription factor CTCF could provide further insight if rs11633032 and rs17191344 can modulate the deduced repressor functions of this transcription factor.

### 4.1 Limitations

We have no data that addresses directly whether or not the Val98Leu change is affecting AnxA2 function. One study [8] has presented preliminary evidence that V98L does not affect the binding affinity of AnXA2 for PCSK9, but it is associated with lower circulating PCSK9 and may be resulting in lower LDL levels. The authors suggest that further studies are needed to examine whether this mutation modifies the function of PCSK9 or has downstream consequences on LDL-R activity. It may be that the coding sequence change could be a site for a transcriptional regulatory element [37], [38]. The sequence around the coding SNP rs17845226 is located in a DNAse I hypersensitive domain, thus it may be a site for positive-acting regulatory sequences, which could interact with intergenic functional SNPs and the *ANXA2* promoter to initiate gene transcription. A second limitation is that it was not possible to impute the genotype in UCLEB for rs17845226, although the examined SNPs were imputed with a reasonable degree of precision (r^2^ = 0.63). Since the SNPs were associated with LDL-C levels in the UCLEB cohort, it is possible that with wet-lab genotyping the effect sizes seen would have been larger, but this imprecision in imputation would is highly unlikely to have resulted in a false positive result.

The *in vitro* data also has limitations. Our data show that a regulatory element near to rs11633032 and rs17191344 SNPs acts as a repressor of *ANXA2* expression in the liver cell line, as it is a major site for LDL-C clearance from the plasma [39], but we could not determine whether or not it also influenced gene expression in other tissues. AnxA2 levels in liver are generally considered low [8], but in line with our studies using HuH7 as model system, stable knockdown of AnxA2 expression in HuH7 resulted in PCSK9 upregulation and a marked reduction in LDL-R levels [40]. However, this may not be representative for all human hepatocellular carcinoma cell lines as AnxA2 depletion in HepG2 cells, which express approximately 5-fold less AnxA2 mRNA compared to HuH7, did not alter PCSK9 or LDL-R protein expression. Moreover, in this study a potentially new mechanism, suggesting a role for AnxA2 in the translational control of PCSK9 protein levels, was proposed [40]. The overall contribution of transcriptional, as described here, and translational regulation of AnxA2 expression affecting PCSK9 maturation and protein levels not only in the extracellular space, but also during PCSK9 synthesis and secretion has yet to be determined *in vivo*.

To demonstrate the functional role of SNP, we used two *in vitro* assays namely EMSA and luciferase, using the hepatoma cell line Huh7, which can only approximate the actual gene expression occurring *in vivo* in the liver, where open chromatin structure and epigenetics have a potential role in gene regulation. In particular, the selected enhancer fragments cannot accurately reflect the natural *in vivo* environment, where chromatin modification and interaction play essential roles in mediating gene expression. We also used the pGL3-promoter vector, since it proved impossible to obtain a cloned sequence of the *ANXA2* promoter due the presence of a repetitive sequence in the promoter which prevented DNA amplification. It is also possible that the enhancer SNPs may affect expression of other distal genes at the locus promoters, although it seems unlikely that alterations in the levels of these genes could be having the effect on LDL-C seen here, with the ASAP data showing no evidence for a strong association of the SNP on expression of other nearby genes.

The *ANXA2* cis-regulatory SNPs, rs11633032, rs17191344 and rs12900101 that we studied have strong LD with 64 SNPs, all in the intergenic region. Here we used ENCODE and a summary tool, HaploReg V4, to select potential functional candidate SNPs. However, the other LD SNPs we did not examine may have a role in transcription regulation. This kind of limitation is unavoidable because there is no conclusive tool to rank likelihood functionality of non-coding variants.

## Conflict of interest

The authors declared they do not have anything to disclose regarding conflict of interest with respect to this manuscript.

## Financial support

RHF is funded by King Abdullah Medical City (KAMC) Holy Capital, and the Ministry of Health, Saudi Arabia (Programme Grant KAMC6). JW is funded by the University College London Genetics Institute which is supported by the National Institute for Health Research, University College London Hospitals Biomedical Research Centre (NIHR UCLHBRC). AZK is funded by the Cardiometabolic Programme within the NIHR UCLHBRC (BRC105CMSH/5982). SEH is a British Heart Foundation Professor and he and JC are funded by a BHF grant (BHFPG08/008) and by the NIHR UCLHBRC. TG acknowledges support from the University of Sydney (U7007). The UCLEB Consortium is supported by a British Heart Foundation Programme Grant (RG/10/12/28456). BRHS is a British Heart Foundation Research Group and is supported by British Heart Foundation (RG/08/013/25942). The WHII study is supported by grants from the Medical Research Council (K013351; ID85374), British Heart Foundation, Stroke Association, National Heart, Lung, and Blood Institute, National Institute on Aging Agency for Health Care Policy and Research, and the John D. and Catherine T. MacArthur Foundation Research Networks on Successful Midlife Development and Socioeconomic Status and Health. Samples from the ELSA DNA Repository (EDNAR), received support under a grant (AG1764406S1) awarded by the National Institute on Ageing (NIA). ELSA was developed by a team of researchers based at the National Centre for Social Research, University College London and the Institute of Fiscal Studies. The data were collected by the National Centre for Social Research. MRC NSHD (MRC1946) is funded by the UK Medical Research Council [MC_UU_12019/1]. BWHHS is supported by funding from the British Heart Foundation and the Department of Health Policy Research Programme (England). EAS is funded by the British Heart Foundation (Programme Grant RG/98002), with Metabochip genotyping funded by a project grant from the Chief Scientist Office of Scotland (Project Grant CZB/4/672). ET2DS is funded by the Medical Research Council (Project Grant G0500877), the Chief Scientist Office of Scotland (Programme Support Grant CZQ/1/38), Pfizer plc (Unrestricted Investigator Led Grant) and Diabetes UK (Clinical Research Fellowship 10/0003985). Research clinics were held at the Wellcome Trust Clinical Research Facility and Princess Alexandra Eye Pavilion in Edinburgh. CAPS was funded by the Medical Research Council and undertaken by the former MRC Epidemiology Unit (South Wales). The DNA bank was established with funding from a MRC project grant. The data archive is maintained by the University of Bristol. The funders had no role in study design, data collection and analysis, decision to publish, or preparation of the manuscript.

## Figures and Tables

**Fig. S10:**
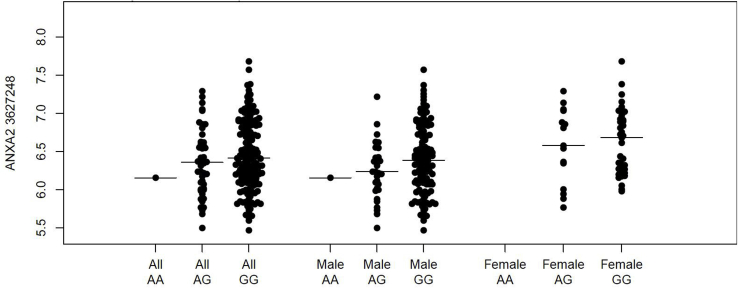


## Appendix A Supplementary data

ANXA2 -Supplementary Data ANXA2 -Supplementary Data

## Footnotes

Appendix A Supplementary data related to this article can be found at http://dx.doi.org/10.1016/j.atherosclerosis.2017.04.010.
