# Supplementary material for: Identifying low density lipoprotein cholesterol associated variants in the Annexin A2 (ANXA2) gene
Source: Atherosclerosis. 2017 Jun;261:60–8. doi: 10.1016/j.atherosclerosis.2017.04.010 (PMC5446264; doi:10.1016/j.atherosclerosis.2017.04.010)
Supplement: ANXA2 -Supplementary Data [file mmc1.docx]

**Identifying Low Density Lipoprotein Cholesterol Associated Variants in the**

***Annexin A2*** **(*ANXA2)*** **Gene**

# Supplementary Data

## Materials and Methods

### Study Cohorts

The Second-Northwick-Park Heart Study (NPHSII) consists of 3,052 (with DNA available n~2700) healthy middle-aged men (50–61 years) who were recruited in 1989 from nine general medical practices in the United Kingdom (UK) and followed for up to 15 years. Information on lifestyle habits, height, weight, blood pressure, and family history of CHD was recorded at baseline and on subsequent follow-ups. Further details about recruitment, measurement, follow-up and incident disease definition have been described elsewhere (1-4). HDL-C was measured using polyethylene glycol 8000 and enzymatic colorimetry on a sample of plasma taken during the sixth year of the study (5).

The UCL-LSHTM-Edinburgh-Bristol (UCLEB) consortium consists of 30,000 participants from 12 well-established UK studies (participants are almost exclusively of European ancestry): Whitehall-II (WHII), British Regional Heart Study (BRHS), English Longitudinal Study of Ageing (ELSA), Medical Research Council National Survey of Health and Development (MRC NSHD), 1958 Birth Cohort (1958BC), Edinburgh Artery Study (EAS), Edinburgh Type 2 Diabetes Study (ET2DS), Edinburgh Heart Disease Prevention Study (EHDPS), Aspirin for Asymptomatic Atherosclerosis Trial (AAAT), Caerphilly Prospective Study (CaPS) and the British Women’s Heart and Health Study (BWHHS). Around 21,000 individuals across the cohorts have been typed using Metabochip, a genotyping platform consisting of about 200,000 SNPs (6). The study extended coverage to approximately 1 million SNPs by imputation of SNPs that have LD r^2^ ≥0.8, where the r^2^ data was referenced to the 1000 Genomes Project. Further details of the consortium can be obtained in Shah et al. (2013).

### Genotyping

The *ANXA2* SNPs rs17845226 and rs17191344 were genotyped in the NPHSII study using Applied Biosystems TaqMan SNP Genotyping Assay. The assay mix was added over 5ng dry DNA and thermocycled as per the manufacturer’s instructions and fluorescence detected with ABI 7900HT. TaqMan genotyping of rs17845266 and rs17191344 was successful with a call rate of ≥90%.

### Statistics

Statistical analysis of NPHSII data was performed using STATA, v14.0 (StataCorp, Texas, USA). Linear regression was used to assess genotypic association with continuous variables (plasma lipid levels) of selective genotypic models. The summary measure for each SNP genotype group was generated based on data disruptions. For normal distributed data, mean and standard division (SD) were calculated. The ANOVA test was applied to either genotypic model (additive or recessive). The Cox proportional hazards model was used to assess time-to-event data, where hazard ratio (HR) and 95% CI were estimated. Stepwise models were used to determine which of the combined genotype groups (1-9) of the rs17191344 and rs17845226 SNPs were having an effect on lipids and CHD. The groups were fitted using 8 dummy variables.  Models used forward selection with a significance level of 5%.

Statistical analysis of UCLEB data and meta-analysis were performed using R version 3.0.2. The studies included in these analyses were: MRC NSHD (MRC1946), WHII, ET2DS, ELSA, EAS, CAPS, BRHS, and BWHHS. The *ANXA2* SNP genotypes for rs11633032 and rs12900101, were imputed (6). Quality of imputation was measured as the ratio of observed (imputed) genetic variance to expected genetic variance (r^2^). The genotypes were phased using MACH1 ([http://csg.sph.umich.edu//abecasis/MaCH/tour/ imputation.html](http://csg.sph.umich.edu//abecasis/MaCH/tour/%20imputation.html)). Missing SNPs were imputed using the 1000 Genomes Project of Europeans as a reference haplotype population, using Minimac software (<http://genome.sph.umich.edu/wiki/Minimac>) (7). For imputed genotypes, a probabilistic model was used in term of “Dose” of a stated allele. In the imputed genotypes, the genotypes include non-integer values in the range 0-2. For recessive genotypic model, the imputed dose ≤ 1.5 was called the genotype 0 (heterozygotes + homozygous major allele) otherwise, the genotype was set to 1 (homozygous minor allele genotype). Patient lipid levels were adjusted to take account of age, sex, the fixed effects of study and genotype. To assess whether there the effect is different between the sexes, we tested the estimated difference between sexes, and no difference was found (Table S10, Supplementary Data).The per-allele effect on the continuous variable was estimated using a linear model, where the variables were log-transformed before analysis due to skewed distribution. We considered a p-value ≤0.05 as significant.

### Bioinformatics

The R1-domain of *ANXA2* is encoded by Exons 4-6, which has eight reported SNPs including one missense variant rs17845226. The latter was selected for further study because it has been validated by HapMap and the 1000 Genome Project, and is the only SNP that has a minor allele frequency (MAF) ≥ 0.05. Multiple algorithms were used to predict the impact of missense mutations (*ANXA2*-R1 rs17845226 SNP V98L) on protein structure and function: Sorting Intolerant Form Tolerant (SIFT), Polyphen-2 V2, and Mutation Assessor V3 (15). The 1000 Genomes Project data and the Broad Institute’s HaploReg V4.1 (16-18) were used to identify variants in strong (r^2^≥0.8) and modest (r^2^≥0.4) linkage disequilibrium (LD) with the *ANXA2*-coding SNP rs17845226. These variants were examined for regulatory annotations from the ENCODE Project (19, 20) and the RoadMap Epigenomics data (21), To visualize variant location, the UCSC Genome Browser was used (22). Criteria for variant selection were based on their location within regulatory element marker peaks including histone modification markers with either promoter or enhancer signatures (defined by H3K4me1 and H3K27Ac as enhancer signs, and H3K4me3 as a promoter sign), DNase I hypersensitivity sites, formaldehyde assisted isolation of regulatory elements (FAIRE), and transcription factor binding sites. The ElDorado tool (Genomatix Software GmbH, Germany) (23) was used to select variants with only strong motif changes (thresholds had core similarity of 1 and matrix similarity of >0.8). The ElDorado data showed that AP-1 and NRSF are predicted to bind to rs11633032 in the presence of the minor allele of the SNP, while MAZR and PLAG1 are predicted to bind to the minor allele of rs12900101.

To assess genotype-gene expression level associations, three expression quantitative trait loci (eQTL) catalogues were used. The first was the Genotype–Tissue Expression (GTEx) portal (24, 25) to calculate the association between rs11633032 genotype and expression of the four closest genes to the SNP in different tissues. The second was the Advanced Study of Aortic Pathology (ASAP) (26).. The third was the eQTL meta-analysis of blood for lipid-regulatory and immune-mediated disease variants (27).

### Cell Culture and preparation of nuclear extracts

The human hepatocellular carcinoma cell line, Huh7, was obtained from the European Collection of Cell Culture (ECACC). The cells were cultured in Dulbecco's Modified Eagle’s Medium (DMEM with 4500 mg/L glucose and L-Glutamine, Sigma Life Sciences) and supplemented with Sigma 10% foetal bovine serum (FBS). Cells were maintained in a 5% CO2 humidified atmosphere at 37°C. Nuclear extract for EMSA was obtained from Huh7 cells as described in (21).

### Electrophoretic Mobility Shift Assay (EMSA)

EMSAs were used to investigate the effect of variants’ genotype on DNA-protein binding. Nuclear extract for EMSA was obtained from hepatocarcinoma Huh7 cells as described in (28). Biotinylated allele-specific probes for the three selected SNPs were incubated with a Huh7 cell nuclear extract (probe sequences in Table S1, Supplementary Data). EMSA was performed as described in (29). Further EMSAs were performed in order to identify proteins that bound to the sequence around the SNP alleles of interest. Rs17191344 SNP was competed with 11 CCCTC-binding factor (CTCF) consensus sequence competitors (30) (CTCF oligo sequences are shown in Table S2, Supplementary Data). Multiplex competitor EMSAs (MC-EMSA) were performed to identify the transcription factors that bind to rs11633032 SNP using seven sets of cocktails, each with ten unlabelled dsDNA consensus sequences for common transcription factors (transcription factors oligo sequences are listed in Table S3 in the Supplementary Data). A cocktail that competed out a binding protein was examined by an additional EMSA, where the individual competitor was incubated separately in order to specify the competing sequence.

## Luciferase Reporter Assay

To generate luciferase-constructs, The *ANXA2* intergenic SNP sequences encompassing the SNP alleles [rs17191344 A>G (776bp) and rs11633032 G>A (593bp) (primer sequences are shown in Table S4, Supplementary Data) were individually inserted into the enhancer site of the pGL3-promoter luciferase reporter vector (Promega) after the SV40 polyadenylation signal according to the manufacturer’s instructions. Both reference allele and alternative allele luciferase-constructs were transfected into Huh7 cells along with the Renilla luciferase pRL-TK as co-transfectant control. The firefly and renilla luciferase activity was detected using Promega’s Dual-Luciferase Reporter Assay System according to the manufacturer’s instructions. Adding a 1520bp fragment of *ANXA2* promoter was impossible due to the presence of a sequence of repetitive elements (1 ALU repeat, and 3 LINE repeats) interspersed in 823bp of the promoter, which was identified using the RepeatMasker software (30).

# Conclusion

In summary, AnxA2 has been previously linked to cholesterol homeostasis and CHD risk via its interaction with PCSK9. We have identified key SNPs in *ANXA2* and its cis-regulatory domain by integrating bioinformatics with disease-association analysis. Although the association study showed the *ANXA2* coding SNP has strong association with LDL-C and CHD risk, the bioinformatics predicted that there is no functional impact of the SNP on the protein. As in many bioinformatics prediction tools, they have a proportion of false negative results. Therefore, further biological functional studies need to verify whether the coding SNP has a role in protein structure or function.

Despite the unknown functional role of *ANXA2* coding SNP, this study has used bioinformatics and functional assays to identify a potential cis-regulatory variant associated with the chromosome 15 locus and in turn with LDL-C levels and CHD risk. The SNPs of modest LD with coded SNP and analysis of expression data suggested *ANXA2* is a potential therapeutic target. The risk allele reduced the expression levels of *ANXA2*, lowered *ANXA2*-mRNA expression and is thus predicted to result in less protein. Low levels of AnxA2 allow PCSK9 to increase LDL-R degradation and thus increase LDL-C levels and risk of CHD.

## Tables

### Table S1: Primer sequences for EMSA probes

| SNP ID | Orientation | Primer Sequence |
| --- | --- | --- |
| Rs17191344 A allele | Forward | TGAGAGCAGGGCC**A**TCGTGGAGGAGTC |
|  | Reverse | GACTCCTCCACGA**T**GGCCCTGCTCTCA |
| Rs17191344 G allele | Forward | TGAGAGCAGGGCC**G**TCGTGGAGGAGTC |
|  | Reverse | GACTCCTCCACGA**C**GGCCCTGCTCTCA |
| Rs12900101 C allele | Forward | TAGGCAGTGCCCCAGCAGGGGCACTGTGCGG |
|  | Reverse | CCGCACAGTGCCCCTGCTGGGGCACTGCCTA |
| Rs12900101 G allele | Forward | TAGGCAGTGCCCCAGGAGGGGCACTGTGCGG |
|  | Reverse | CCGCACAGTGCCCCTCCTGGGGCACTGCCTA |
| Rs11633032 G allele | Forward | TGAAGCAGCTCAGACGGCAAGCGAAGGGCAA |
|  | Reverse | TTGCCCTTCGCTTGCCGTCTGAGCTGCTTCA |
| Rs11633032 A allele | Forward | TGAAGCAGCTCAGACAGCAAGCGAAGGGCAA |
|  | Reverse | TTGCCCTTCGCTTGCTGTCTGAGCTGCTTCA |
| Rs116255697 A allele | Forward | GTTCAAAGTACCACAAATCTCTAAGGCAGGG |
|  | Reverse | CCCTGCCTTAGAGATTTGTGGTACTTTGAAC |
| Rs116255697 G allele | Forward | GTTCAAAGTACCACAGATCTCTAAGGCAGGG |
|  | Reverse | CCCTGCCTTAGAGATCTGTGGTACTTTGAAC |

### Table S2: Primer sequences for CTCF isoforms probes

| Oligo ID | Orientation | Oligo Sequence |
| --- | --- | --- |
| CTCF-1 | Forward | CCAGCCTTCAGCAGGTGGCACTGTTG |
|  | Reverse | CAACAGTGCCACCTGCTGAAGGCTGG |
| CTCF-2 | Forward | TGTTTCAGCCACCTGCTGGGCAGCAG |
|  | Reverse | CTGCTGCCCAGCAGGTGGCTGAAACA |
| CTCF-3 | Forward | CAGCCCTGCCCCCTGCCGGGCCCTGTG |
|  | Reverse | CACAGGGCCCGGCAGGGGGCAGGGCTG |
| CTCF-4 | Forward | ATGATGGCCAGCAGAGGGCGCATGGC |
|  | Reverse | ATGATGGCCAGCAGAGGGCGCATGGC |
| CTCF-5 | Forward | AGACAGCGCCCTCTGTTGGAATTAGCC |
|  | Reverse | GGCTAATTCCAACAGAGGGCGCTGTCT |
| CTCF-6 | Forward | CTGATTTCCACTAGGGGGCCGTGGTG |
|  | Reverse | CACCACGGCCCCCTAGTGGAAATCAG |
| CTCF-7 | Forward | CAACGGCGCCCCTTCGTGGTGATAAT |
|  | Reverse | ATTATCACCACGAAGGGGCGCCGTTG |
| CTCF-8 | Forward | TCCTGGCCCCCTCTGCTGGACATCCT |
|  | Reverse | AGGATGTCCAGCAGAGGGGGCCAGGA |
| CTCF-9 | Forward | CCAAGGCTGCCCCTGCTGGCAAAGGC |
|  | Reverse | GCCTTTGCCAGCAGGGGCAGCCTTGG |
| CTCF-10 | Forward | AGCTACACCGACAGGGGGCGCCAGCC |
|  | Reverse | GGCTGGCGCCCCCTGTCGGTGTAGCT |
| CTCF-11 | Forward | CTTGAGTGCCCTCTGGTGGGCAATAG |
|  | Reverse | CTATTGCCCACCAGAGGGCACTCAAG |

### Table S3: Consensus sequence for multiplex competitor EMSA

F and R refer to oligonucleotides orientation Forward and Reverse respectively

| **Oligo name** | **consensus sequence** |
| --- | --- |
| AP1_F | CGCTTGATGACTCAGCCGGAA |
| AP1_R | TTCCGGCTGAGTCATCAAGCG |
| AP2a_F | GATCGAACTGACCGCCCGCGGCCCGT |
| AP2a_R | ACGGGCCGCGGGCGGTCAGTTCGATC |
| AR_F | GAAGTCTGGTACAGGGTGTTCTTTTTG |
| AR_R | CAAAAAGAACACCCTGTACCAGACTTC |
| Brn3_F | CACAGCTCATTAACGCGC |
| Brn3_R | GCGCGTTAATGAGCTGTG |
| CBP_F | AGACCGTACGTGATTGGTTAATCTCTT |
| CBP_R | AAGAGATTAACCAATCACGTACGGTCT |
| CDP_F | ACCCAATGATTATTAGCCAATTTCTGA |
| CDP_R | TCAGAAATTGGCTAATAATCATTGGGT |
| CEBP_F_R | TGCAGATTGCGCAATCTGCA |
| cMyb_F | TACAGGCATAACGGTTCCGTAGTGA |
| cMyb_R | TCACTACGGAACCGTTATGCCTGTA |
| CREB_F | AGAGATTGCCTGACGTCAGAGAGCTAG |
| CREB_R | CTAGCTCTCTGACGTCAGGCAATCTCT |
| CTCF_F | GGCGGCGCCGCTAGGGGTCTCTCT |
| CTCF_R | AGAGAGACCCCTAGCGGCGCCGCC |
| E2F1_F | ATTTAAGTTTCGCGCCCTTTCTCAA |
| E2F1_R | TTGAGAAAGGGCGCGAAACTTAAAT |
| Egr_F | GGATCCAGCGGGGGCGAGCGGGGGCGA |
| Egr_R | TCGCCCCCGCTCGCCCCCGCTGGATCC |
| ER_F | GGATCTAGGTCACTGTGACCCCGGATC |
| ER_R | GATCCGGGGTCACAGTGACCTAGATCC |
| Ets_F | GGGCTGCTTGAGGAAGTATAAGAAT |
| Ets_R | ATTCTTATACTTCCTCAAGCAGCCC |
| Ets1_F | GATCTCGAGCAGGAAGTTCGA |
| Ets1_R | TCGAACTTCCTGCTCGAGATC |
| FAST1_F | TGTGTATTCA |
| FAST1_R | TGAATACACA |
| GAS_F | AAGTACTTTCAGTTTCATATTACTCTA |
| GAS_R | TAGAGTAATATGAAACTGAAAGTACTT |
| GATA_F | CACTTGATAACAGAAAGTGATAACTCT |
| GATA_R | AGAGTTATCACTTTCTGTTATCAAGTG |
| Gfi1_F | TAAATCACTGC |
| Gfi1_R | GCAGTGATTTA |
| GR_F | AGAGGATCTGTACAGGATGTTCTAGAT |
| GR_R | ATCTAGAACATCCTGTACAGATCCTCT |
| HIF1a_F | TCTGTACGTGACCACACTCACCTC |
| HIF1a_R | GAGGTGAGTGTGGTCACGTACAGA |
| ISRE_F | AAGTACTTTCAGTTTCATATTACTCTA |
| ISRE_R | TAGAGTAATATGAAACTGAAAGTACTT |
| HNF4_F | CTCAGCTTGTACTTTGGTACAACTA |
| HNF4_R | TAGTTGTACCAAAGTACAAGCTGAG |
| IRF1_F | GGAAGCGAAAATGAAATTGACT |
| IRF1_R | AGTCAATTTCATTTTCGCTTCC |
| MEF1_F | GATCCCCCCAACACCTGCTGCCTGA |
| MEF1_R | TCAGGCAGCAGGTGTTGGGGGGATC |
| MEF2_F | GATCGCTCTAAAAATAACCCTGTCG |
| MEF2_R | CGACAGGGTTATTTTTAGAGCGATC |
| MIBP1_F | TCTTTTCCCA |
| MIBP1_R | TGGGAAAAGA |
| MycMax_F_R | GGAAGCAGACCACGTGGTCTGCTTCC |
| NF1_F | TTTTGGATTGAAGCCAATATGATAA |
| NF1_R | TTATCATATTGGCTTCAATCCAAAA |
| NFE2_F | TGGGGAACCTGTGCTGAGTCACTGGAG |
| NFE2_R | CTCCAGTGACTCAGCACAGGTTCCCCA |
| NFATc_F | CGCCCAAAGAGGAAAATTTGTTTCATA |
| NFATc_R | TATGAAACAAATTTTCCTCTTTGGGCG |
| NFkB_F | AGTTGAGGGGACTTTCCCAGGC |
| NF-kB_R | GCCTGGGAAAGTCCCCTCAACT |
| NR5A2_F | GATCAACGACCGACCTTGAG |
| NR5A2_R | CTCAAGGTCGGTCGTTGATC |
| OCT1_F | TGTCGAATGCAAATCACTAGAA |
| OCT1_R | TTCTAGTGATTTGCATTCGACA |
| p53_F | TACAGAACATGTCTAAGCATGCTGGGG |
| p53_R | CCCCAGCATGCTTAGACATGTTCTGTA |
| Pax5_F | GAATGGGGCACTGAGGCGTGACCACCG |
| Pax5_R | CGGTGGTCACGCCTCAGTGCCCCATTC |
| Pbx1_F | CTCCAATTAGTGCATCAATCAATTCG |
| Pbx1_R | CGAATTGATTGATGCACTAATTGGAG |
| Pit1_F | TGTCTTCCTGAATATGAATAAGAAATA |
| Pit1_R | TATTTCTTATTCATATTCAGGAAGACA |
| PPAR_F | AGGTCAAAGGTCA |
| PPAR_R | TGACCTTTGACCT |
| PR_F | GATCCTGTACAGGATGTTCTAGCTACA |
| PR_R | TGTAGCTAGAACATCCTGTACAGGATC |
| RAR_F | AGGGTAGGGTTCACCGAAAGTTCACTC |
| RAR_R | GAGTGAACTTTCGGTGAACCCTACCCT |
| RXR_F | AGCTTCAGGTCAGAGGTCAGAGAGCT |
| RXR_R | AGCTCTCTGACCTCTGACCTGAAGCT |
| SIE_F | GTGCATTTCCCGTAAATCTTGTCTACA |
| SIE_R | TGTAGACAAGATTTACGGGAAATGCAC |
| Smad_F | GTCTAGACCA |
| Smad_R | TGGTCTAGAC |
| Smad34_F | TCGAGAGCCAGACAAAAAGCCAGACATTTAGCCAGACAC |
| Smad34_R | GTGTCTGGCTAAATGTCTGGCTTTTTGTCTGGCTCTCGA |
| Smuc_F | GGATCCCCCAACACCTGCTGCCTGA |
| Smuc_R | TCAGGCAGCAGGTGTTGGGGGATCC |
| Sp1_F | ATTCGATCGGGGCGGGGCGAGC |
| Sp1_R | GCTCGCCCCGCCCCGATCGAAT |
| SRE_F | GGATGTCCATATTAGGACATCT |
| SRE_R | AGATGTCCTAATATGGACATCC |
| Stat1_F | CATGTTATGCATATTCCTGTAAGTG |
| Stat1_R | CACTTACAGGAATATGCATAACATG |
| Stat3_F | GATCCTTCTGGGAATTCCTAGATC |
| Stat3_R | GATCTAGGAATTCCCAGAAGGATC |
| Stat4_F | GAGCCTGATTTCCCCGAAATGATGAGC |
| Stat4_R | GCTCATCATTTCGGGGAAATCAGGCTC |
| Stat5_F | AGATTTCTAGGAATTCAATCC |
| Stat5_R | GGATTGAATTCCTAGAAATCT |
| Stat56_F | GTATTTCCCAGAAAAGGAAC |
| Stat56_R | GTTCCTTTTCTGGGAAATAC |
| Tbet_F_R | AATTTCACACCTAGGTGTGAAATT |
| TFE3_F | GATCTGGTCATGTGGCAAGGC |
| TFE3_R | GCCTTGCCACATGACCAGATC |
| TFEB_F | CACGTG |
| TFEB_R | CACGTG |
| TFIID_F | GCAGAGCATATAAAATGAGGTAGGA |
| TFIID_R | TCCTACCTCATTTTATATGCTCTGC |
| TGIF_F | ACTCTGCCTGTCAAGCGAGG |
| TGIF_R | CCTCGCTTGACAGGCAGAGT |
| TR_F | AGCTTCAGGTCACAGGAGGTCAGAGAG |
| TR_R | CTCTCTGACCTCCTGTGACCTGAAGCT |
| USF1_F | CACCCGGTCACGTGGCCTACACC |
| USF1_R | GGTGTAGGCCACGTGACCGGGTG |
| VDR_F | AGCTTCAGGTCAAGGAGGTCAGAGAGC |
| VDR_R | GCTCTCTGACCTCCTTGACCTGAAGCT |
| YY1_F | CGCTCCCCGGCCATCTTGGCGGCTGGT |
| YY1_R | ACCAGCCGCCAAGATGGCCGGGGAGCG |
| ZEB_F | GATCTGGCCAAAGGTGCAGGATC |
| ZEB_R | GATCCTGCACCTTTGGCCAGATC |
| HNF1_F | GTTAATGATTAAC |
| HNF1_R | GTTAATCATTAAC |
| ARP1_F | AGGTGACCTTTGCCCA |
| ARP1_R | TGGGCAAAGGTCACCT |
| NFY_F | ATCAGCCAATCAGAGC |
| NFY_R | GCTCTGATTGGCTGAT |
| HNF3_F | GCCCATTGTTTGTTTTAAGCC |
| HNF3_R | GGCTTAAAACAAACAATGGGC |
| BARP_F | TCACTCAAGTTCAAGTTATT |
| BARP_R | AATAACTTGAACTTGAGTGA |
| SREBP1_F | TTTGAAAATCACCCCATGCAAACTC |
| SREBP1_R | GAGTTTGCATGGGGTGATTTTCAAA |
| HSF1_F | GATCTCGGCTGGAATATTCCCGACCTGGCAGCCGA |
| HSF1_R | TCGGCTGCCAGGTCGGGAATATTCCAGCCGAGATC |

### Table S4: Cloning primers for luciferase sequence fragment

Primers were designed to incorporate restriction enzymes (underlined) and pGL3- promoter vector site (highlighted)

| SNP ID | Restriction Enzyme | Orientation | Primer Sequence | Fragment Size |
| --- | --- | --- | --- | --- |
| rs11633032 | BamH I  Sal I | Forward | 5’-AAATCGATAAGGATCCTGTGTGCTCTTTGGTTCAAGG-3’ | 585bp |
|  |  | Reverse | 5’-AAGGGCATCGGTCGACCACCTCAGCCTCCCAAGTAG-3’ |  |
| rs17191344 | Sal I | Forward | 5’-ATAAGGATCCGTCGACAACCACTTCAGAGGCGATCA-3’ | 767bp |
|  |  | Reverse | 5’-AAGGGCATCGGTCGACAGGCACTCACCCAAAGAAGT-3’ |  |

### Table S5: The Second-Northwick-Park Heart Study (NPHSII) cohort baseline characteristics:

|  | Value (SD) | Range | IQR |
| --- | --- | --- | --- |
| Sample size | 2463 |  |  |
| Age | 56.1 (3.4) | 49-64 | 53-59 |
| Smoker (%) | 1694 (68.8) |  |  |
| Body mass index (kg/m^2^) | 26.5 (3.5) | 16.6-49.5 | 24.2-28.4 |
| Type 2 diabetes (%) | 0 | - | - |
| Systolic blood pressure (mmHg) | 138.7 (19.2) | 90-219.5 | 125-150 |
| Diastolic blood pressure (mmHg) | 84.6 (11.2) | 48.5-135 | 77-92 |
| TC ( mmol/l) | 5.75 (1.01) | 2.6-10 | 5-6.4 |
| LDL-C ( mmol/l) | 3.10 (1.00) | 0.1-7.6 | 2.4-3.8 |
| HDL-C ( mmol/l) | 0.80 (0.24) | 0.25-1.94 | 0.66-0.98 |
| TG* ( mmol/l) | 1.72 (0.53) | 1.24 – 2.6 | 0.2-0.9 |

Mean and standard deviation (SD), where appropriate, are shown. * Analysis carried out on log transformed and median and IQR presented.

.

### Table S6: Stepwise regression analysis of Total Cholesterol, LDL-cholesterol and CHD in subjects with different combined of rs17191344 and rs17845226: Second-Northwick-Park Heart Study (NPHSII)

| Group | TC  B (se) | P-value | LDL-C  B (se) | *P*-value | CHD  HR (95% CI) | P-value |
| --- | --- | --- | --- | --- | --- | --- |
| Reference  GG/AA  Reference  GG/CC | 0  0.482 (0.221)  R2=0.002 | 0.029 | 0  0.649 (0.244)  R2=0.004 | 0.008 | -  -  -  1.00  6.53 (1.62-26.27) | 0.008 |

Adjusted for age and practice

For TC and LDL-C, only those with two copies of the minor allele of both SNPs differed significantly from the group that carried the major alleles of both SNPs (AA/CC), and for the analysis above the reference group used is the combination of AA/CC and all other groups. For CHD, only those with the genotype GG/CC differed significantly from the group that carried the major alleles of both SNPs (AA/CC), and for this analysis the reference group used is the combination of AA/CC and all other groups.

### Table S7: The UCL-LSHTM-Edinburgh-Bristol (UCLEB) consortium: Total number of individuals, CHD rates and baseline characteristics for incident CHD in the seven studies.

|  | BRHS | BWHHS | CaPS | EAS | ELSA | ET2DS | WHII | MRC NSHD 1946 |
| --- | --- | --- | --- | --- | --- | --- | --- | --- |
| Number included in  analysis | 2387 | 1922 | 1381 | 850 | 1934 | 1049 | 3311 | 2139 |
| Age, years | 68.9 (5.61) | 71.5 (5.28) | 61.90 (5.05) | 64.46 (5.64) | 73.64 (9.55) | 67.90 (4.20) | 49.00 (5.96) | 50 (0) |
| Sex, % male | 100 | 0 | 100 | 49 | 53 | 52 | 76 | 50 |
| BMI, kg/m2 | 26.83 (3.63) | 27.67 (4.99) | NA | 6.20 (1.10) | 27.48 (4.49) | 31.39 (5.66) | 25.21 (3.54) | 27.63 (4.63) |
| TC, mmol/l | 6.36 (1.03) | 6.62 (1.22) | 6.20 (1.10) | 7.08 (1.33) | 5.71 (1.28) | 4.31 (0.90) | 6.44 (1.12) | 6.09 (1.07) |
| HDL-C, mmol/l | 1.15 (0.24) | 1.62 (0.45) | NA | 1.45 (0.38) | 1.49 (0.39) | 1.29 (0.36) | 1.41 (0.40) | 1.67 (0.52) |
| LDL-C, mmol/l | 3.89 (1.00) | 4.14 (1.10) | NA | 5.33 (1.22) | 3.42 (1.06) | NA | 4.37 (1.00) | 3.52 (0.97) |
| TG*, mmol/l | 2.05 (1.22) | 1.91 (1.05) | 1.87 (1.10) | 1.53 (0.90) | 1.80 (1.11) | NA | 1.46 (1.15) | 2.16 (1.51) |
| SBP,mmHg | 144.0 (19.95) | 150.0 (25.36) | 145.0 (22.32) | NA | 138.9 (19.63) | 133.3 (16.46) | 120.5 (13.14) | 136.2 (20.01) |
| DBP, mmHg | 81.8 (12.80) | 80.1 (11.76) | 81.7 (11.98) | NA | 73.0 (11.46) | 69.1 (9.00) | 79.8 (9.12) | 84.6 (12.17) |

Mean and standard deviation (SD), where appropriate, are shown. * analysis carried out on log transformed and median and IQR presented.

The British Regional Heart Study (BRHS), British Women’s Heart and Health Study (BWHHS), the Caerphilly Prospective Study (CaPS), the Edinburgh Artery Study (EAS), the English Longitudinal Study of Aging (ELSA), the Edinburgh Type 2 Diabetes Study (ET2DS), Whitehall II study (WHII), and MRC National Survey of Health and Development 1946 (MRC NSHD 1946).

### Table S8: Imputation quality of UCLEB consortium

Quality of imputation was measured as the ratio of observed (imputed) genetic variance to expected genetic variance (r^2^) in 8 studies

| SNP | WHII | BRHS | BWHHS | ET2DS | EAS | CAPS | MRC1946 | ELSA |
| --- | --- | --- | --- | --- | --- | --- | --- | --- |
| rs11633032 | 0.63 | 0.64 | 0.63 | 0.63 | 0.62 | 0.64 | 0.63 | 0.63 |
| rs12900101 | 0.63 | 0.63 | 0.63 | 0.63 | 0.62 | 0.64 | 0.63 | 0.63 |

### Table S9: An association of *ANXA2* intergenic SNPs and lipid traits in the UCLEB Consortium

|  | AnxA2 SNP | Lipid trait | Effect Size^a^ | Std. Error | p-Value^a^ | p-Value^b^ | Number of samples by genotype |
| --- | --- | --- | --- | --- | --- | --- | --- |
| Men | **rs12900101** | TC | 0.147 | 0.086 | 0.088 | 0.505 | CC=5995, CG=2824, GG=161 |
|  |  | LDL-C | 0.188 | 0.090 | 0.036 | 0.323 | CC=4626, CG=2190, GG=124 |
|  |  | HDL-C | 0.010 | 0.031 | 0.734 | 0.814 | CC=4998, CG=2361, GG=131 |
|  |  | Log TG | -0.007 | 0.047 | 0.876 | 0.833 | CC=5084, CG=2419, GG=134 |
|  | **rs11633032** | TC | 0.171 | 0.087 | 0.049 | 0.505 | GG=5992, AG=2824, AA =158 |
|  |  | LDL-C | 0.215 | 0.091 | 0.018 | 0.328 | GG=4626, AG=2193, AA =121 |
|  |  | HDL-C | 0.009 | 0.031 | 0.774 | 0.800 | GG=5000, AG=2362, AA =128 |
|  |  | Log TG | -0.002 | 0.048 | 0.968 | 0.846 | GG=5086, AG=2420, AA =131 |
| Women | **rs12900101** | TC | 0.092 | 0.126 | 0.461 | 0.158 | CC=3714, CG=1773, GG=90 |
|  |  | LDL-C | 0.104 | 0.119 | 0.383 | 0.089 | CC=3302, CG= 1588, GG=84 |
|  |  | HDL-C | 0.020 | 0.045 | 0.668 | 0.778 | CC=3682, CG=1760, GG=90 |
|  |  | Log TG | 0.410 | 0.052 | 0.427 | 0.642 | CC= 3374, CG= 1626, GG=85 |
|  | **rs11633032** | TC | 0.105 | 0.127 | 0.410 | 0.153 | GG=3718, GA=1771, AA=88 |
|  |  | LDL-C | 0.104 | 1.120 | 0.390 | 0.084 | GG=3306, GA= 1586, AA=82 |
|  |  | HDL-C | 0.029 | 1.046 | 0.532 | 0.783 | GG=3686, GA=1758, AA=88 |
|  |  | Log TG | 0.044 | 1.052 | 0.401 | 0.654 | GG= 3378, GA= 1624 , AA=83 |
| Men & Women | **rs12900101** | TC | 0.117 | 0.072 | 0.104 | 0.116 | CC=9709, CG=4597, GG=251 |
|  |  | LDL-C | 0.143 | 0.072 | 0.048 | 0.046 | CC=7928, CG=3778 , GG=208 |
|  |  | HDL-C | 0.016 | 0.026 | 0.536 | 0.716 | CC=8680, CG=4121, GG=221 |
|  |  | Log TG | 0.007 | 0.035 | 0.836 | 0.965 | CC=8458, CG=4045, GG=219 |
|  | **rs11633032** | TC | 0.139 | 0.073 | 0.056 | 0.114 | GG=9716, AG=4595, AA =246 |
|  |  | LDL-C | 0.160 | 0.073 | 0.029 | 0.045 | GG=7932, AG=3779, AA =203 |
|  |  | HDL-C | 0.019 | 0.026 | 0.468 | 0.710 | GG=8686, AG=4120, AA =216 |
|  |  | Log TG | 0.013 | 0.036 | 0.726 | 0.968 | GG=8464, AG=4044, AA =214 |

a *p*-value for corresponding recessive model

^a^Effect size is the main effect of the homozygous minor allele genotype on lipid concentration estimated in a linear model containing age and study ID as covariates.

*^b^ p*-value for corresponding additive model.

### Table S10: The estimated difference between sexes of *ANXA2* intergenic SNPs and lipid traits in UCLEB Consortium

| SNP ID | Lipid trait | Mean | Standard error | *p*-value |
| --- | --- | --- | --- | --- |
| rs12900101 | LDL-C | 0.084 | 0.102 | 0.82 |
|  | TC | 0.054 | 0.103 | 0.522 |
| rs11633032 | LDL-C | 0.11 | 0.104 | 0.29 |
|  | TC | 0.066 | 0.104 | 0.52 |

To assess whether there the effect of the SNP on TC and LDL-C was different between the sexes, values for these traits were estimated as described in supplementary methods section of statistics, but separately in men and women. The estimates were compared using a t-test. The question of interaction of sex with genotype was addressed by adding an interaction term to the regression model, thus estimating the effects of genotype, sex and genotype: sex on lipid fraction. In all cases the interaction term was not significant (both in terms of its estimated effect and in terms of the proportion of variance explained.

## Figure legends

### Figure S1: The *ANXA2* exon 6 amino acid conservation sequence in different species.

Schematic presentation of the ANXA2 exon-6 amino acid sequence in different species (<https://genome-euro.ucsc.edu>). The *ANXA2* coding SNP rs17845226 (Valine 98 Leucine) is highly conserved across species.

### Figure S2: Diagram of chromosome 15 locus

The diagram shows the *ANXA2* and nearby genes. Single nucleotide polymorphisms (SNPs) selected for the study are shown: one coding SNP rs1785226 and two intergenic SNPs showing modest LD, rs17191344 and rs11633032.

### Figure S3: *ANXA2* rs17191344 linkage disequilibrium (LD) plot.

A LD plot was generated using SNAP V2.2 (Broad Institute; https://www.broad institute.org/mpg/snap/ldplot.php). The LD between the query SNP and each corresponding proxy SNP are represented by diamonds, the query SNP (rs17191344) is represented by a red diamond, while LD SNPs are represented by orange diamonds. r-squared value shown on the left Y-axis, where r2≥0.8 indicates a strong LD, and r2≤0.2 indicates a weak LD. A blue line indicates estimated recombination rates and their value are presented on the right Y-axis. LD and recombination rates are based on HapMap Phase II (CEU, YRI and JPT+CHB). CEU= Utah residents with Northern and Western European ancestry from the CEPH collection.

### Figure S4: Combined genotype association between rs17191344 and rs17845226 SNPs genotype and LDL-C levels in the NPHSII cohort

### Figure S5: The average of the phenotype by genotype class for AnxA2 intergenic SNPs in the UCLEB study.

The Genotype is presented as a binary character: class 0 (homozygous major allele) dose 0 - 0.5, class 2 (homozygous minor allele) dose 1.5 – 2, and class 1 (heterozygotes) dose 0.5-1.5. The error bars are the 95% confidence interval for the mean.

### Figure S6: DNA binding and expression of the transcription factors of the *ANXA2-* inergenic SNP rs11633032.

MC-EMSA analysis. Nuclear proteins from the Huh7 cell line were incubated with 7 cocktails of unlabelled DNA competitors (70 well-characterized DNA-binding proteins) for 15 minutes, then a 5’ end-biotinylated allele-specific probe was added. The multiplex competitors compete out any specific interactions with a labelled probe, eliminating or reducing any positive shift result. (A) *ANXA2*- intergenic SNP rs11633032 MC-EMSA for G allele of the SNP, allele specific bands indicated by arrows were eliminated by cocktail 1. (B) The single competitors from cocktail 1 (a) were run individually in a further EMSA, showing Egr1 and GATA resulted in competition (eliminated bands indicated by dotted arrows).

### Figure S7: CTCF-Specific Protein Binding to *ANXA2* intergenic SNP rs17191344 in Huh7 Cells.

1. CTCF consensus sequence used in EMSA was compared with genomic sequence around rs17191344. The single nucleotide polymorphism G allele matches more closely with the consensus sequence, which may increase the binding affinity to CTCF, suggesting CTCF may be the protein binding here. (B) EMSA analysis of the *ANXA2*- intergenic SNP rs17191344 G allele which is competed out by 11 isoforms of CTCF. The G allele specific bands, indicated by arrows, were eliminated by at least 3 isoforms of CTCF (eliminated bands indicated by dotted arrows).

### Figure S8: *ANXA2* expression by rs11633032 genotype.

Data and graph from Genotype–Tissue Expression (GTEx) Portal. The tissue-specific association between the rs11633032 and genotype and expression level of *ANXA2* in three different tissues (liver, whole blood, and coronary artery) were investigated. Although no significant association was found due to sample size, the SNP genotype showed the same expression trend in all three tissues, where carriers of the minor alleles have lower gene expression.

### Figure S9: *ANXA2* Gene expression by rs11633032 genotype in liver in the ASAP study

Additive linear models used to detect the association between SNP and gene expression levels. Gene expression measurement in a patient with rs11633032 genotype, which can be AA, AG and GG. Expression data is shown for the whole sample, men and women.

## References

1. Miller G, Bauer K, Barzegar S, Cooper J, Rosenberg R. Increased activation of the haemostatic system in men at high risk of fatal coronary heart disease. Thrombosis and haemostasis. 1996;75(5):767-71.

2. Hawe E, Talmud P, Miller G, Humphries S. Family history is a coronary heart disease risk factor in the Second Northwick Park Heart Study. Annals of Human Genetics. 2003;67(2):97-106.

3. Cooper JA, Miller GJ, Bauer KA, Morrissey JH, Meade TW, Howarth DJ, et al. Comparison of Novel Hemostatic Factors and Conventional Risk Factors for Prediction of Coronary Heart Disease. Circulation. 2000;102(23):2816-22.

4. Miller G, Bauer K, Barzegar S, Foley A, Mitchell J, Cooper J, et al. The effects of quality and timing of venepuncture on markers of blood coagulation in healthy middle-aged men. Thrombosis and haemostasis. 1995;73(1):82-6.

5. Nanjee MN, Cooke CJ, Wong JS, Hamilton RL, Olszewski WL, Miller NE. Composition and ultrastructure of size subclasses of normal human peripheral lymph lipoproteins: quantification of cholesterol uptake by HDL in tissue fluids. Journal of lipid research. 2001;42(4):639-48.

6. Shah T, Engmann J, Dale C, Shah S, White J, Giambartolomei C, et al. Population Genomics of Cardiometabolic Traits: Design of the University College London-London School of Hygiene and Tropical Medicine-Edinburgh-Bristol (UCLEB) Consortium. PloS one. 2013;8(8):e71345.

7. Howie B, Fuchsberger C, Stephens M, Marchini J, Abecasis GR. Fast and accurate genotype imputation in genome-wide association studies through pre-phasing. Nature genetics. 2012;44(8):955-9.

8. Reva B, Antipin Y, Sander C. Predicting the functional impact of protein mutations: application to cancer genomics. Nucleic acids research. 2011.

9. Ward LD, Kellis M. HaploReg: a resource for exploring chromatin states, conservation, and regulatory motif alterations within sets of genetically linked variants. Nucleic acids research. 2012;40(D1):D930-D4.

10. Ward LD, Kellis M. HaploReg v4: systematic mining of putative causal variants, cell types, regulators and target genes for human complex traits and disease. Nucleic acids research. 2015.

11. The1000GenomesProjectConsortium. An integrated map of genetic variation from 1,092 human genomes. Nature. 2012;491(7422):56-65.

12. TheEncodeProjectConsortium. A User's Guide to the Encyclopedia of DNA Elements (ENCODE). PLoS Biology. 2011;9(4):e1001046.

13. TheEncodeProjectConsortium. An Integrated Encyclopedia of DNA Elements in the Human Genome. Nature. 2012;489(7414):57-74.

14. Bernstein BE, Stamatoyannopoulos JA, Costello JF, Ren B, Milosavljevic A, Meissner A, et al. The NIH roadmap epigenomics mapping consortium. Nature biotechnology. 2010;28(10):1045-8.

15. Zweig AS, Karolchik D, Kuhn RM, Haussler D, Kent WJ. UCSC genome browser tutorial. Genomics. 2008;92(2):75-84.

16. Cartharius K, Frech K, Grote K, Klocke B, Haltmeier M, Klingenhoff A, et al. MatInspector and beyond: promoter analysis based on transcription factor binding sites. Bioinformatics. 2005;21(13):2933-42.

17. Lonsdale J, Thomas J, Salvatore M, Phillips R, Lo E, Shad S, et al. The Genotype-Tissue Expression (GTEx) project. Nat Genet. 2013;45(6):580-5.

18. GTExConsortium. The Genotype-Tissue Expression (GTEx) pilot analysis: Multitissue gene regulation in humans. Science (New York, NY). 2015;348(6235):648-60.

19. Folkersen L, Hooft Fvt, Chernogubova E, Agardh HE, Hansson GK, Hedin U, et al. Association of Genetic Risk Variants With Expression of Proximal Genes Identifies Novel Susceptibility Genes for Cardiovascular Disease. Circulation: Cardiovascular Genetics. 2010;3(4):365-73.

20. Westra H-J, Peters MJ, Esko T, Yaghootkar H, Schurmann C, Kettunen J, et al. Systematic identification of trans eQTLs as putative drivers of known disease associations. Nat Genet. 2013;45(10):1238-43.

21. Boardman-Pretty F, Smith AJ, Cooper J, Palmen J, Folkersen L, Hamsten A, et al. Functional analysis of a carotid intima-media thickness locus implicates BCAR1 and suggests a causal variant. Circulation: Cardiovascular Genetics. 2015;8(5):696-706.

22. Kim TH, Abdullaev ZK, Smith AD, Ching KA, Loukinov DI, Green RD, et al. Analysis of the vertebrate insulator protein CTCF-binding sites in the human genome. Cell. 2007;128(6):1231-45.

23. Tarailo-Graovac M, Chen N. Using RepeatMasker to Identify Repetitive Elements in Genomic Sequences. Current Protocols in Bioinformatics: John Wiley & Sons, Inc.; 2002.
